# Preventing the clinical manifestations and disease progression of coronavirus disease using clinically proven protease inhibitors

**DOI:** 10.1186/s13054-020-03235-4

**Published:** 2020-08-18

**Authors:** Tomoya Sagawa, Ken-ichiro Inoue, Hirohisa Takano

**Affiliations:** 1 Department of Environmental Engineering, Graduate School of Engineering, Kyoto University, Kyoto, Japan; 2 Inflammation and Immunology, Graduate School of Medical Science, Kyoto Prefectural University of Medicine, Kyoto, Japan; 3 School of Nursing, University of Shizuoka, Shizuoka, Japan; 4 Graduate School of Global Environmental Studies, Kyoto University, C Cluster, Katsura Campus, Nishikyo-ku, Kyoto, 615-8540 Japan

To the Editor:

Among the patients infected with severe acute respiratory syndrome coronavirus 2 (SARS-CoV-2), older adults and those predisposed to cardiovascular or respiratory diseases are particularly vulnerable to severe complications, including acute respiratory distress syndrome (ARDS), multiple organ failure, and disseminated intravascular coagulation (DIC), all of which need critical care [1]. The production and application of effective and safe vaccines and/or drugs worldwide will require a substantial amount of time and cost. Thus, medically preventing the coronavirus disease (COVID-19) using the existing safe and mass-produced medicines is the need of the hour, especially to protect vulnerable populations.

Doi et al. reported that a combination treatment of favipiravir and nafamostat mesylate, a protease inhibitor, may be effective for critically ill patients with COVID-19, possibly via blockade of virus entry and replication as well as inhibition of hypercoagulopathy [2].

Not only to treat but also to decrease the number of critically ill patients with COVID-19, preventing the disease progression and clinical manifestation of COVID-19 is essential. Here, we additionally propose the prophylactic use of clinically proven protease inhibitors based on the following clinical and experimental evidence.

First, protease inhibitors, such as camostat mesylate and nafamostat mesylate, have long been used in the Japanese clinical practice with safe clinical outcomes. Second, these drugs suppress the entry of SARS-CoV-2 into cells via the angiotensin-converting enzyme 2 and transmembrane protease serine 2 [2, 3]. Third, nafamostat mesylate, an anticoagulant, has been widely used for extracorporeal circulation and in patients with DIC in Japan. Furthermore, thromboembolic events among hospitalized patients with COVID-19 have seen an increase, and anticoagulants could improve in-hospital mortality and survival [4]. Thus, protease inhibitors could also prevent an unfavorable prognosis of COVID-19, especially in cases with associated coagulopathies.

Finally, we demonstrated that the urinary trypsin inhibitor (UTI), another protease inhibitor, protects against systemic inflammation, ARDS, multiple organ failure, and DIC [5]. UTI prevents the accelerated expression of systemic inflammatory cytokines, which can contribute to the amelioration of the cytokine storm, a critical factor for the unfavorable prognosis of COVID-19.

Taken together, these clinically proven protease inhibitors can serve as promising prophylactics to prevent the manifestation and progression of COVID-19, by blocking the entry of SARS-CoV-2 and inhibiting subsequent inflammation, coagulopathies, and multiple organ failure in vulnerable populations, especially those who interact with patients with COVID-19, until vaccines and/or drugs suitable for the prevention and/or treatment of the disease are readily available worldwide.

## Data Availability

Not applicable.

## Abbreviations

SARS-CoV-2: Severe acute respiratory syndrome coronavirus 2
COVID-19: Coronavirus disease
ARDS: Acute respiratory distress syndrome
DIC: Disseminated intravascular coagulation
UTI: Urinary trypsin inhibitor
